# Louisville Medical College

**Published:** 1850-11

**Authors:** 


*Louisville Medical College.*—Prof. Paul F. Eve, of Augusta, Ga., has been appointed to the Chair of Surgery, in this Institution, made vacant by the resignation of Prof. Gross. The College is fortunate in the selection. We have not learned who succeeds Prof. Eve in the Georgia Medical College.

